# Cumulative ecological risk and problematic smartphone use among Chinese college students: the roles of performance goal orientation, learning goal orientation and psychological resilience

**DOI:** 10.3389/fpsyg.2024.1467653

**Published:** 2024-12-24

**Authors:** Jinliang Guan, Peng Yu, Chengzhen Liu, Wangyan Ma

**Affiliations:** ^1^School of Teacher Education, Chaohu University, Hefei, China; ^2^School of Foreign Languages, Chaohu University, Hefei, China; ^3^Faculty of Psychology, Southwest University, Chongqing, China; ^4^Faculty of Language and Literature, Anhui Sanlian University, Hefei, China

**Keywords:** cumulative ecological risk, performance goal orientation, learning goal orientation, psychological resilience, problematic smartphone use

## Abstract

Problematic smartphone use (PSU) has emerged as a pressing concern among college students, with cumulative ecological risk identified as a crucial yet enigmatic contributor. To unravel its underlying mechanisms, we devised and validated a model that delves into the mediating roles of performance and learning goal orientations, as well as the moderating influence of psychological resilience. Our investigation encompassed 2011 Chinese college students spanning from freshmen to seniors, aged 16 to 25, utilizing comprehensive scales to assess various constructs. The findings revealed that both performance and learning goal orientations serve as pivotal mediators in the relationship between cumulative ecological risk and PSU. Furthermore, psychological resilience was found to moderate not only the direct link between cumulative ecological risk and PSU but also the indirect pathway through learning goal orientation. These insights underscore the practical significance of fostering healthy achievement goals and enhancing psychological resilience among college students, thereby mitigating the prevalence of PSU.

## 1 Introduction

With the growing development of science and technology and the deep integration of mobile communication and traditional Internet, the information era dominated by mobile Internet has arrived. Mobile Internet has both the characteristics of traditional Internet, such as openness, equality and interaction, and the advantages of convenience, timeliness and individuation. As an important carrier of mobile Internet, smartphones play an important role nowadays. The 51th Statistical report on China’s Internet Development released by China Internet Network Information Center (2023) showed that the number of Mobile Internet users in China had reached 1.065 billion by December 2022. Among them, the age group between 10 and 29 years old accounted for 28.5%. College students have a high incidence of problematic smartphone use (PSU). Studies showed that the detection rate of PSU among college students is 25–40%, and college students spend an average of 6 h on their smartphones (Chen et al., 2017; Wang et al., 2014). The convenience of smartphones can enable individuals to complete tasks with less effort and improve efficiency. But at the same time, overuse of smartphones can lead to PSU (Ahn and Jung, 2016). PSU makes it difficult for college students to concentrate in class, reduces happiness in life, and can even lead to social anxiety, personality disorders, and suicide attempts (Billieux et al., 2015; Bragazzi and Puente, 2014; Dietz and Henrich, 2014; Volkmer and Lermer, 2019). Meanwhile, PSU can also harm individual’s mental health (Błachnio and Przepiorka, 2019), cause physical discomfort (Salunke and Shah, 2019), and even threaten the life safety of college students (Hersh et al., 2019).

Environmental factors, such as family, school, peers and society, are important factors affecting PSU among college students. Risk factors in family, school, peers and society, such as family functioning, school belonging, teacher-student relationships, friendship quality, and social support, all increase the risk of PSU among college students (Jin et al., 2021; Li and Lv, 2022; Mansourian et al., 2014; Shi et al., 2020; Zhen et al., 2019). However, most of the researchers investigated the influence of a single risk factor, and few studied the effect of cumulative risk. In real life, individuals have to face the superimposed influence of multiple risk factors. It is impossible to accurately grasp the internal law of PSU among college students only from the perspective of a single factor (Wang et al., 2012). Therefore, combining multiple factors of individual development and examining PSU through cumulative ecological risk can help us find out the reasons affecting PSU among college students more comprehensively.

In terms of cumulative ecological risks and problematic mobile phone use, most studies focus on middle school students (Liu et al., 2022). Liu et al. (2022) showed that the cumulative social environmental risk index had a positive predictive effect on the four forms of mobile phone addiction of middle school students (mobile social network addiction, mobile game addiction, mobile information acquisition addiction and mobile short video addiction). In addition, the cumulative risk index showed a stronger predictive effect in predicting all four types of phone addiction compared to a single environmental factor. However, there is a lack of research on the impact of cumulative ecological risk on problematic mobile phone use among college students. Furthermore, the mechanism of the impact of cumulative ecological risk on problematic mobile phone use among college students is also unclear. On this basis, the effects of cumulative ecological risk on college students’ PSU and its mechanism are discussed.

### 1.1 Cumulative ecological risk and PSU

Cumulative risk model shows that risk factors existing in different ecological subsystems, such as family, school, peers and society, exert an impact on individual psychology and behavior in a superimposed manner (Lengua et al., 2008). In theory, the effect of multiple risk factors is often stronger than that of a single risk factor. If multiple risk factors add up, individuals are likely to be more harmed (Masten, 2014). In fact, after incorporating the cumulative risk index into the regression equation, the researchers found that cumulative ecological risk had a more significant predictive effect on adolescent Internet addiction than a single ecological risk (Li et al., 2016). In addition, in the face of the impact of multiple risk factors, the effect of intervention for only one factor will be reduced (Evans et al., 2013).

Johnson and Puplampu (2008) proposed the eco-technology-microsystem theory. This theory shows that eco-technology microsystem is a microsystem in the whole development ecosystem of an individual, reflecting the influence of family, school, peers and social environment factors on the use of individual electronic products. For example, from the perspective of family, the study of Shi et al. (2020) shows that good family functions can improve college students’ peer trust level and reduce their dependence on mobile phones, thus promoting them to obtain a sense of psychological happiness. From the perspective of school, college students with a higher sense of belonging to school have a lower level of PSU (Jin et al., 2021). From the perspective of peers, college students with poor friendship quality are more dependent on mobile phones (Li and Lv, 2022). From a social perspective, low social support can have a positive impact on college students’ mobile phone dependence (Wei, 2012).

Previous researchers mainly focused on the relationship between cumulative ecological risk and problematic mobile phone use and problematic Internet use among adolescents (Li et al., 2016; Liu et al., 2022; Luo et al., 2017). There is a lack of research on cumulative ecological risk and PSU among college students. In addition, with the popularity of smartphones, more and more college students use smartphones to surf the Internet, so it is necessary to investigate the ecological risk factors affecting PSU among college students.

### 1.2 The mediating role of performance goal orientation and learning goal orientation

Although cumulative ecological risk may lead to PSU, the association between cumulative ecological risk and PSU may be indirect (Li et al., 2016). Li et al. (2016) showed the influence of cumulative ecological risk on adolescents’ Internet addiction and the parallel mediating role of basic psychological need satisfaction and positive outcome expectation. As an important driving force, basic psychological need is an individual psychological variable and an internal need. In addition to internal needs, the achievement goal orientation of this study also includes external incentives, that is, external stimuli that can cause individual behavior or the value of external stimuli obtained by individuals through learning. Motivated behavior is a continuous behavior under the joint action of internal needs and external conditions. Eccles et al. (1998) proposed the expected value theory of achievement motivation, which holds that individual achievement behavior is influenced by multiple factors. Individual’s choice of achievement behavior is influenced by the expectation of success and the value of goal and task. In this theory, factors such as family, school, peers and society in ecological risk can exert a certain influence on performance goal orientation and learning goal orientation as environmental factors (Wright and Masten, 2005).

According to the cognitive-behavior model proposed by Davis (2001), individuals with poor achievement goals have lower self-efficacy (Sitzmann and Yeo, 2013), and low self-efficacy as a kind of maladaptive cognition is a proximal factor of pathological Internet use (Davis, 2001). Studies had shown that students with poor mastery of goals in performance goal orientation and learning goal orientation are prone to mobile phone dependence (Yang and Xu, 2018). Furthermore, college students addicted to mobile phones are more sensitive and worried about situations that may cause failure, and are easy to make negative expectations about possible failure and adopt a defensive attitude toward failure. This suggests that people with low achievement motivation avoid failure in a maladaptive way (mobile phone addiction) (Jia, 2009). On the one hand, Internet addicts have impaired limbic system function, which is related to learning motivation and inhibition (Nie et al., 2016). On the other hand, in order to obtain more educational opportunities, students strive to achieve academic success. When their development needs for competence, intimacy and autonomy are not satisfied, students may have dissatisfaction with the school and the thought of retreat, which may lead to excessive use of the Internet (Ryan and Deci, 2000). The severity of mobile phone addiction, as a subcategory of Internet addiction, may be high (Kwon et al., 2013; Nielsen and Fjuk, 2010).

According to the achievement motivation theory proposed by Button et al. (1996), human behaviors in specific environments (such as mobile phone addiction) often have corresponding motivation bases. This motivation usually involves two aspects, one is the internal need, which is the psychological tendency of the organism to feel a certain lack and strive to obtain satisfaction, and is the internal power to promote the behavior. The other is the external incentive, which is the external stimulus that can cause individual behavior or the recognition of the value of the external stimulus obtained by the individual through learning. Motivated behavior is carried out continuously under the combined force of internal needs and external conditions. And performance goal orientation and learning goal orientation are just such internal needs. Ecological risks will lead to insufficient achievement goals, and then PSU. In summary, our research delves into the pivotal mediating functions of performance goal orientation and learning goal orientation, elucidating their role in shaping the relationship between cumulative ecological risk and PSU among college students.

### 1.3 The moderating role of psychological resilience

First, as a personality trait, psychological resilience refers to the phenomenon that individuals can successfully cope with and recover from major pressure or danger (Tugade and Fredrickson, 2004). Kumpfer (1999) proposed the “individual-process-environment” psychological resilience theoretical model, revealing that people, environment and adaptation results interact and influence each other. The model posits that when confronted with a stressor or challenge, an intricate interplay occurs between the individual and their environment. Simultaneously, a constellation of internal resilience factors, spanning cognitive, emotional, mental, physical, and behavioral domains, exerts a vital influence, either facilitating successful coping or contributing to maladjustment. The interaction between internal resilience factors (resilience) and environment (ecological risk) helps individuals successfully cope with maladaptive factors (e.g., PSU), and resilience may play an important role as a buffer between ecological risk and mobile phone addiction.

Previous studies had found that psychological resilience is a protective factor of college students’ mobile phone dependence, and psychological resilience can significantly negatively predict college students’ mobile phone dependence (Huang et al., 2019). More importantly, psychological resilience has an important impact on individual cognition and emotion, and can regulate individual perception in situational events (Fu and Wang, 2019). College students with high levels of psychological resilience are better at using their own cognitive resources to regulate their mental health status, so as to buffer the adverse effects of ecological risks on Internet addiction (Huang, 2018; Huang, 2020; Wu, 2018). Based on the above, the moderating effect of psychological resilience on the relationship between cumulative ecological risk and PSU is explored.

Second, the “individual-process-environment” psychological resilience theoretical model believes that internal factors of psychological resilience include the individual’s adaptation to external environment and the process of actively changing external environment (Kumpfer, 1999). When confronted with stressors or challenges, psychological resilience can help individuals cope successfully. Studies had shown that lack of achievement goal motivation leads to increased individual pressure (Podsakoff et al., 2007). At this time, internal psychological resilience factors can reduce the occurrence of risky behaviors (such as mobile phone addiction) and promote individuals to develop well. In addition, the resilience theory believes that individuals with high psychological resilience can effectively cope with inadequate performance goal orientation and learning goal orientation (Fergus and Zimmerman, 2005). A study has shown that psychological resilience has an important impact on individual cognition and emotion, and can regulate individual perception in situational events (Fu and Wang, 2019). College students with high levels of psychological resilience are better at using their cognitive resources to cope with stress, and thus buffer Internet addiction caused by stress (Huang, 2018; Huang, 2020; Wu, 2018). Furthermore, psychological resilience is a protective factor for mobile phone addiction (Huang et al., 2019). Based on the above, we explored the moderating effects of psychological resilience on the relationship between performance goal orientation and PSU, as well as the relationship between learning goal orientation and PSU.

### 1.4 The current study

**Figure 1:**
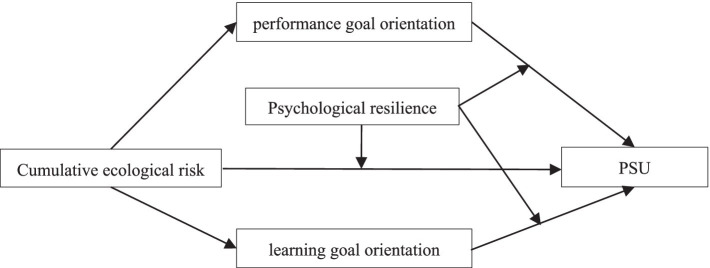
The hypothetical model.

Drawing upon prior studies, we have formulated a hypothetical model (Figure 1) that aims to test the mediating roles of performance goal orientation and learning goal orientation, as well as the moderating influence of psychological resilience. This model offers a nuanced understanding of the intricate relationship between cumulative ecological risk and PSU among college students. It not only elucidates the mechanisms underlying how cumulative ecological risk impacts PSU but also reveals the conditions under which this effect may be intensified or mitigated. Consequently, we propose the following hypotheses:

*Hypothesis 1*. Cumulative ecological risk is positively correlated with PSU.

*Hypothesis 2*. Performance goal orientation and learning goal orientation play mediating roles in the relationship between cumulative ecological risk and PSU.

*Hypothesis 3*. Psychological resilience moderates the relationship between cumulative ecological risk and PSU.

*Hypothesis 4*. Psychological resilience moderates the relationship between performance goal orientation and PSU, as well as the relationship between learning goal orientation and PSU.

## 2 Method

### 2.1 Participants and procedure

Participants were selected using the convenience sampling method. Students ranging from freshmen to seniors were recruited from 8 undergraduate universities located in Hunan Province, Anhui Province, and Guangxi Zhuang Autonomous Region. The recruited participants must have used smartphones. Before collecting data, we obtained informed consent from the students. A well-trained psychology graduate student explained the principles of voluntary participation and confidentiality to the students during class. We collected data using pen and paper surveys on the spot. It took approximately 20 min for each student to fill out the questionnaire anonymously. Upon completion of the questionnaire, each student received a small gift as a token of our gratitude.

First, the participants were provided with instructions outlining the purpose of the test, the method of answering questions, and the principle of anonymity. Second, the participants were instructed to fill out the questionnaire truthfully. A total of 2,125 paper questionnaires were distributed, and 2011 valid questionnaires were received, resulting in an effective recovery rate of 94.64%. Since the missing values accounted for less than 5% and were of the scoring type variable, they were replaced with the average value. The participants were between 16 and 25 years old (*M* = 19.89; *SD =* 1.32). There were 1,145 male students (56.94%) and 866 female students (43.06%). There were 678 freshmen (33.72%), 497 sophomores (24.71%), 450 juniors (22.38%) and 386 seniors (19.19%).

### 2.2 Measures

#### 2.2.1 Cumulative ecological risk

First, based on the eco-technology-microsystem theory proposed by Johnson and Puplampu (2008), and previous studies in the field of cumulative ecological risk by Gerard and Buehler (2004), four risk factors related to mobile phone addiction were comprehensively selected from four ecological subsystems: family, school, peers, and society, to measure cumulative ecological risk.

Second, the selection principles (systematicness, representativeness, adaptability, development and feasibility) of ecological risk factors proposed by Li et al. (2016) are relatively comprehensive and reasonable. Therefore, based on this principle and the characteristics of problematic smartphone use among college students, this study selected four ecological risk factors, namely, family function (family risk), school belonging (school risk), friendship quality (peer risk) and social support (social risk).

Third, referring to the statistical method of Bakker et al. (2010), the scale scores of each risk factor were standardized, all Z-scores were added, and the total cumulative risk index was obtained after reverse scoring. The higher the score, the more serious the degree of multiple risk factors experienced by an individual.

##### 2.2.1.1 Family functioning scale

The Chinese adaptation of the Family Closeness and Adaptability Scale (FACESII-CV), originally developed by Olson et al. (1982) and subsequently revised by Fei et al. (1991), was employed. Comprising a total of 30 questions (e.g., “When confronted with difficulties, family members strive to support each other”), the scale assesses two key dimensions: family closeness and family adaptability. A 5-point Likert scale was utilized, with 1 representing “never” and 5 indicating “always.” A higher score on this scale signifies better family functioning. Prior research on college students has demonstrated the good reliability and validity of this scale (Deng and Zheng, 2012). The Cronbach’s *α* of this scale was 0.89 in the current study.

##### 2.2.1.2 School belonging scale

The Psychological Sense of School Membership (PSSM) scale, originally developed by Goodenow (1993) and subsequently translated and adapted by Pan et al. (2011), was utilized. Comprising a total of 18 questions (for instance, “I feel like a member of this school”), the scale encompasses three dimensions: sense of belonging, identity, and school attachment. A 5-point Likert scale was employed, with 1 representing “never” and 5 signifying “always.” A higher score on this scale indicates a stronger sense of school belonging. Prior research on college students has established the good reliability and validity of this scale (Pan et al., 2011). The Cronbach’s *α* of this scale was 0.89 in the current study.

##### 2.2.1.3 Friendship quality scale

The Friendship Quality Scale, originally compiled by Furman and Buhrmester (1985) and later revised by Fan and Fang (2004), was employed. It comprises a total of 15 questions (e.g., “When you need to do something, do your friends often help you?”), encompassing four dimensions: help and support, friend conflict, partnership, and intimacy. A 5-point Likert scale was utilized, with 1 indicating “never” and 5 signifying “always.” Higher scores on this scale reflect better friendship quality. Prior research on college students has demonstrated the good reliability and validity of this scale (Fan and Fang, 2004). The Cronbach’s *α* of this scale was 0.73 in the current study.

##### 2.2.1.4 Perceived social support scale

The Chinese version of the Perceived Social Support Scale (PSSS), adapted from Dahlem et al. (1991) and further revised by Jiang (1999), was utilized. It comprises 12 questions [such as “Some people (teachers, classmates, and relatives) will be with me when I encounter problems”] that measure three dimensions of social support: family support, friend support, and other support. Taking into account the unique context of college students and drawing upon the research of Yan and Zheng (2006), the “leaders, relatives, and colleagues” category under the “other support” dimension in the original scale was modified to “teachers, classmates, and relatives.” A 7-point Likert scale was employed, with 1 representing “strongly disagree” and 7 signifying “strongly agree.” Higher scores on this scale indicate a greater perceived level of social support. Prior research on college students has established the good reliability and validity of this adapted scale (Yan and Zheng, 2006). The Cronbach’s *α* of this scale was 0.94 in the current study.

#### 2.2.2 Achievement goal oriented questionnaire

The Achievement Goal Orientation Questionnaire, initially compiled by Button et al. (1996) and subsequently translated and revised by Xu et al. (2000), was employed. It consists of 12 questions (e.g., “I am willing to do tasks that enable me to learn new things”) that assess two dimensions: performance goal orientation and learning goal orientation. A 7-point Likert scale was utilized, with 1 indicating “strongly disagree” and 7 signifying “strongly agree.” A higher score on this questionnaire reflects a stronger achievement goal orientation. Prior research conducted on college students has demonstrated the good reliability and validity of this questionnaire (Xu et al., 2000). The Cronbach’s *α* of performance goal orientation dimension was 0.79, and the Cronbach’s α of learning goal orientation dimension was 0.90 in the current study.

#### 2.2.3 Psychological resilience

The Psychological Resilience Scale, originally compiled by Connor and Davidson (2003) and subsequently translated and revised by Yu and Zhang (2007), was utilized. This scale comprises 25 items (such as “I will not be discouraged by failures”) that measure three key dimensions of psychological resilience: toughness, self-improvement, and optimism. A 5-point Likert scale was employed, with 1 representing “very inconsistent” and 5 signifying “very consistent.” Higher scores on this scale indicate a higher level of psychological resilience. Prior research conducted on college students has validated the good reliability and validity of this scale (Jia and Wang, 2018). The Cronbach’s α of the scale was 0.93 in the current study.

#### 2.2.4 Smartphone addiction scale for college students

The smartphone addiction scale for college students compiled by Su et al. (2014) was adopted. This scale consists of 22 items (e.g., “Procrastination due to smartphone use causes a lot of trouble for me.”), including 6 dimensions: withdrawal behavior, highlighting behavior, social comfort, negative impact, App use and App update. A 5-point scale was used, with 1 meaning “strongly disagree” and 5 meaning “strongly agree.” The higher the score, the more serious the individual’s degree of PSU. This questionnaire has shown good reliability and validity in previous studies on college students (Su et al., 2014). The Cronbach’s *α* of the scale was 0.91 in the current study.

### 2.3 Data analysis

The collected data were analyzed utilizing SPSS 26.0. Initially, descriptive statistics were computed to summarize the variables, and Pearson correlation coefficients were calculated to assess the relationships between them. Subsequently, Model 4 of the PROCESS macro developed by Hayes (2013) was employed to examine the mediating effects of performance goal orientation and learning goal orientation on the relationship between cumulative ecological risk and PSU. Additionally, Model 15 of the PROCESS macro was utilized to test the moderating influence of psychological resilience on this relationship.

## 3 Results

### 3.1 Common method bias

To assess the potential for common method bias in the data, the Harman single factor method was employed. The results revealed the presence of 13 factors with eigenvalues exceeding 1, and the variance explained by the first factor amounted to 20.45%. This value falls below the critical threshold of 40%, suggesting that the data were not significantly impacted by common method bias.

### 3.2 Descriptive statistics and correlation analysis

According to the classification criteria of SAS-C score groups by Zhang et al. (2016), those with score > =77 are considered as addiction groups. There were 157 addicts, accounting for 7.66% of the respondents. A correlation analysis is performed on the data, and the results are summarized in Table 1, which presents the mean, standard deviation, and correlation matrix of the key variables. The findings indicate that cumulative ecological risk displays a significant negative correlation with both performance goal orientation and learning goal orientation, as well as with psychological resilience. Conversely, it shows a significant positive correlation with PSU. Furthermore, performance goal orientation and learning goal orientation are significantly positively correlated with psychological resilience but negatively correlated with PSU. Lastly, a significant negative correlation is observed between psychological resilience and PSU.

**Table 1:** Means, standard deviations and correlations of the study variables.

	*M*	*SD*	1	2	3	4	5
1 Cumulative ecological risk	−0.07	3.03	1				
2 Performance goal orientation	4.87	0.87	−0.49**	1			
3 Learning goal orientation	5.10	0.95	−0.58**	0.70**	1		
4 Psychological resilience	3.67	0.49	−0.59**	0.37**	0.47**	1	
5 PSU	2.69	0.58	0.41**	−0.34**	−0.41**	−0.41**	1

*N* = 2011. **p < 0.01.

### 3.3 Cumulative ecological risk and its sub-risks and PSU

In terms of the method of data analysis of existing studies, we first put each ecological risk factor into the regression analysis separately, and then put it into the regression analysis together with the cumulative ecological risk without this ecological risk factor. The predictive effect of cumulative ecological risk and a single risk factor on college students’ PSU was explained by comparing the change of regression coefficient before and after the two conditions (MacKenzie et al., 2011).

As can be seen from Table 2, by comparing the size of regression coefficients, three of the originally significantly ecological risk factors (family risk, school risk and social risk) still had significantly predictive effects. However, any risk factor was a smaller predictor of PSU than cumulative ecological risk. Therefore, cumulative ecological risk factors were stronger than a single risk factor in predicting PSU.

**Table 2:** Predictive effects of ecological risk factors on PSU (before and after controlling for the total number of other ecological risk factors).

	Before controlling for the total number of other ecological risk factors			After controlling for the total number of other ecological risk factors		
	*b*	*SE*	*β*	*b*	*SE*	*β*
Family risk	0.34	0.02	0.33***	0.18	0.03	0.18***
School risk	0.37	0.02	0.36***	0.23	0.03	0.22***
Peer risk	0.22	0.02	0.22***	−0.03	0.02	−0.03
Social risk	0.36	0.02	0.35***	0.16	0.03	0.16***
Cumulative ecological risk	*β =* 0.41***					

***p < 0.001.

### 3.4 Testing for mediation

Initially, all variables were standardized to ensure comparability, and gender was coded using dummy variables, where 1 represented boys and 0 represented girls. Subsequently, Model 4 of the PROCESS macro created by Hayes (2013) was utilized to investigate the mediation effect. In view of the fact that studies had shown that gender and age factors have an important impact on individual mobile phone use (Chen and Kim, 2013; Ryan et al., 2014). Therefore, in the process of data analysis, these two variables were included in the control variables.

The outcomes of the mediation analysis, with performance goal orientation serving as the mediator, are presented in Table 3. The results indicate that cumulative ecological risk significantly and positively predicts PSU (*β* = 0.41, *p* < 0.001). Additionally, cumulative ecological risk negatively predicts both performance goal orientation (*β* = −0.48, *p* < 0.001) and learning goal orientation (*β* = −0.57, *p* < 0.001). Furthermore, both performance goal orientation (*β* = −0.07, *p* < 0.05) and learning goal orientation (*β* = −0.23, *p* < 0.001) negatively predict PSU. The mediation analysis reveals that performance goal orientation mediates the relationship between cumulative ecological risk and PSU, with a mediating effect of 0.03. This effect is statistically significant, as evidenced by the 95% Bootstrap confidence interval [0.01, 0.06], contributing 7.32% to the total effect. Similarly, learning goal orientation also mediates this relationship, with a larger mediating effect of 0.13 (95% Bootstrap CI [0.09, 0.17]), accounting for 31.71% of the total effect.

**Table 3:** Testing the mediating effect.

Pathway	Effect	95%CI	
		Low	High
Cumulative ecological risk → PSU (direct effect)	0.25	0.20	0.30
Cumulative ecological risk → Performance goal orientation → PSU	0.03	0.01	0.06
Cumulative ecological risk → Learning goal orientation → PSU	0.13	0.09	0.17
Cumulative ecological risk → PSU (total effect)	0.41	0.37	0.45

### 3.5 Testing for moderation

Following the moderation model testing methodology advocated by Wen and Ye (2014), we incorporated controls for gender and age factors. Subsequently, we utilized the PROCESS program developed by Hayes (2013) to empirically evaluate the moderation model.

The detailed analysis results are presented in Table 4. We delved into the moderation effects of psychological resilience on the direct and indirect pathways linking cumulative ecological risk, performance goal orientation, and PSU. In Equation 1, cumulative ecological risk emerged as a significant negative predictor of performance goal orientation (*β* = −0.48, *p* < 0.001). Similarly, in Equation 2, cumulative ecological risk significantly negatively predicted learning goal orientation (*β* = −0.57, *p* < 0.001). Moving on to Equation 3, cumulative ecological risk had a significant direct effect on PSU (*β* = 0.17, *p* < 0.001), and this relationship was further moderated by psychological resilience, as evidenced by the significant interaction term (*β* = −0.10, *p* < 0.001). Performance goal orientation also significantly predicted PSU (*β* = −0.07, *p* < 0.05), yet the interaction with psychological resilience did not reach statistical significance (*β* = −0.02, *p* > 0.05). Conversely, learning goal orientation significantly predicted PSU (*β* = −0.17, *p* < 0.001), and this relationship was moderated by psychological resilience, as indicated by the significant interaction term (*β* = −0.08, *p* < 0.01). In summary, psychological resilience moderates both the direct relationship between cumulative ecological risk and PSU and the indirect relationship between learning goal orientation and PSU.

**Table 4:** Testing the moderating effect.

Independent variables	Equation 1: PGO			Equation 2: LGO			Equation 3: PSU		
	*β*	*SE*	*t*	*β*	*SE*	*t*	*β*	*SE*	*t*
Gender	−0.16	0.04	−4.06***	−0.25	0.04	−6.57***	−0.08	0.04	−1.99*
Age	−0.02	0.01	−1.44	−0.02	0.01	−1.55	−0.03	0.01	−1.84
Cumulative ecological risk	−0.48	0.02	−24.34***	−0.57	0.02	−31.26***	0.17	0.03	6.21***
Psychological resilience							−0.21	0.03	−8.32***
Cumulative ecological risk × Psychological resilience							−0.10	0.02	−4.35***
PGO							−0.07	0.03	−2.47*
PGO × Psychological resilience							−0.02	0.03	−0.62
LGO							−0.17	0.03	−5.69***
LGO × Psychological resilience							−0.08	0.03	−2.60**
*R^2^*	0.24			0.35			0.25		
*F*	213.65***			360.81***			74.19***		

PGO, performance goal orientation; LGO, learning goal orientation. *p < 0.05, **p < 0.01, ***p < 0.001.

**Figure 2:**
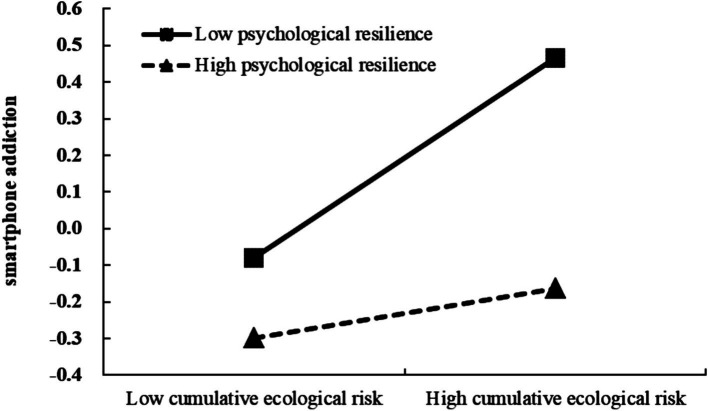
The moderating effect of psychological resilience on the relationship between cumulative ecological risk and PSU.

In order to further analyze the moderating effect trend of psychological resilience, psychological resilience was divided into high and low groups by adding and subtracting one standard deviation from the average value for simple slope analysis (Figure 2). Cumulative ecological risk significantly negatively predicted PSU in the low psychological resilience group (*β*_simple_ = 0.27, *t* = 7.04, *p* < 0.001). In the high psychological resilience group, the negative predictive effect of cumulative ecological risk on PSU was weakened (*β*_simple_ = 0.07, *t* = 2.04, *p* < 0.05).

**Figure 3:**
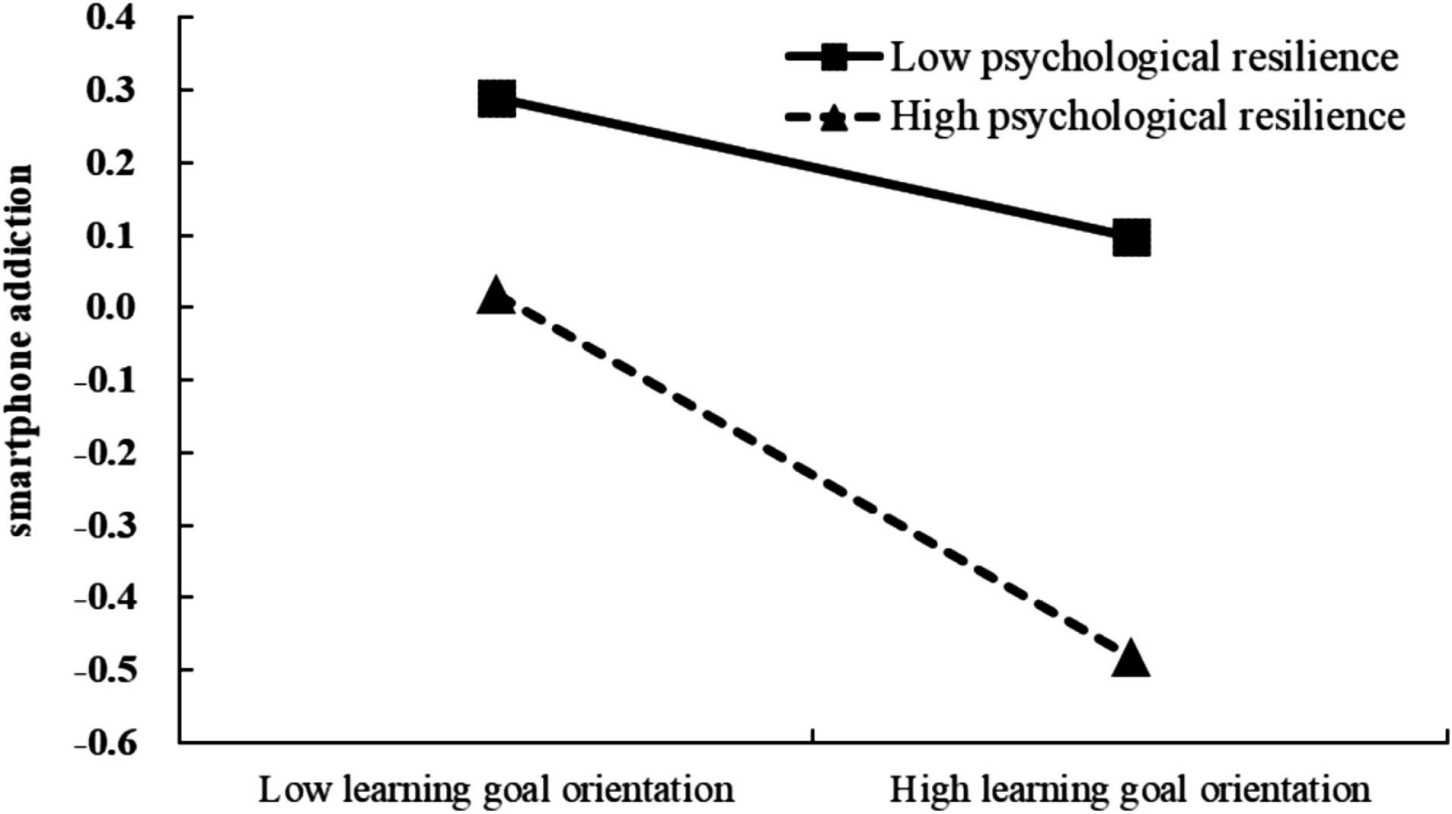
The moderating effect of psychological resilience on the relationship between learning goal orientation and PSU.

As shown in Figure 3, learning goal orientation had a significantly predictive effect on PSU in the low psychological resilience group (*β*_simple_ = −0.10, *t* = −2.12, *p* < 0.05). In the high psychological resilience group, the negative predictive effect of learning goal orientation on PSU was enhanced (*β*_simple_ = −0.25, *t* = −6.21, *p* < 0.001).

## 4 Discussion

A moderated mediation model was formulated to explore the complex interplay between cumulative ecological risk, PSU, and the mediating mechanisms of performance goal orientation and learning goal orientation. Additionally, the model examined the moderating influence of psychological resilience on both the direct and indirect pathways connecting cumulative ecological risk to PSU.

### 4.1 Cumulative ecological risk and PSU

The results show that cumulative ecological risk has a stronger predictive effect on college students’ PSU than a single risk, which is consistent with a previous study (Li et al., 2016). Therefore, it is more valuable to study the effect of cumulative ecological risk on PSU than a single risk factor. The results of existing studies have shown that cumulative ecological risk and Internet addiction of college students appear a “positive acceleration pattern” (Farrell et al., 1992; Forehand et al., 1998), that is, the cumulative total effect of all risk factors is greater than the sum of the effects of all risk factors. When risk factors appear alone, they may not have a decisive effect. Only under the combined influence of family, school, peer and social risks, risk factors will become more threatening to individuals.

The results also show that cumulative ecological risk can positively predict PSU, and Hypothesis 1 is verified. This result supports the eco-technology-microsystems theory that for college students, support from family, school, peers and society is the key to their healthy physical and mental development. When there are multiple factors in the environment that are not conducive to the healthy physical and mental development of individuals, individuals will not be able to develop healthily, so they seek other environments, such as the Internet, to meet their needs (Masten, 2014). A previous study has demonstrated that the cumulative social environmental risk index can accurately forecast four distinct forms of mobile phone addiction, including addiction to mobile social networks, mobile games, mobile information acquisition, and mobile short videos, all of which are positively influenced by trait mindfulness. Moreover, the cumulative risk index predicted the four types of mobile phone addiction better than any single environmental factor (Liu et al., 2022).

### 4.2 The mediating role of performance goal orientation and learning goal orientation

Another finding is that performance goal orientation and learning goal orientation play mediating roles in the relationship between cumulative ecological risk and PSU, and Hypothesis 2 is verified. This result supports the expected value theory of achievement motivation, that is, family, school, peers, society and other factors in ecological risk, as environmental factors of individual achievement motivation, have certain influences on performance goal orientation and learning goal orientation (Wright and Masten, 2005). Studies have shown that family, school and peers factors have a joint effect on the learning motivation of college students. Specifically, good family environment, positive teacher-student relationship and peer cooperative learning have a significant impact on learning results (Zhao, 2017).

This result also supports the cognitive-behavioral model, that is, students with poor performance goal orientation and learning goal orientation are more likely to become dependent on mobile phones (Yang and Xu, 2018). Some studies have found that some college students addicted to the Internet had excellent grades before entering college, but they failed to adjust their roles in time after entering college, resulting in a certain sense of frustration. Internet use can help individuals achieve successful satisfaction in the Internet world (Shen et al., 2004). According to the theory of achievement motivation, motivation is an internal need derived from internal psychological factors. When internal motivation is insufficient, people often need to use the Internet to escape from reality (Button et al., 1996). The result of the current study supports previous studies that people with lower achievement goals have higher degrees of Internet addiction (Niemz et al., 2005), thus making PSU with similar symptoms and behavioral defects to Internet addiction, more serious (Jeong et al., 2016).

The result showed that the total variance explained by the performance goal orientation mediation is slight (7.32%). The possible reason is that performance goal-oriented individuals avoid challenges. When performance is reduced in the face of obstacles, they attribute their failure to poor ability, resulting in less guilt and less negative emotions (Elliott and Dweck, 1988), which in turn leads to a smaller mediating effect. In addition, a study has shown that students with poor mastery of goals in achievement goal orientation are more likely to become dependent on mobile phones (Yang and Xu, 2018). In other words, students with poor learning goal orientation are more likely to have mobile phone dependence, which leads to larger mediating effect of learning goal orientation compared with performance goal orientation.

### 4.3 The moderating effect of psychological resilience

The results show that psychological resilience could moderate both the relationship between cumulative ecological risk and PSU, and the relationship between learning goal orientation and PSU. Hypothesis 3 is verified, and Hypothesis 4 is partially verified.

First, psychological resilience moderated the relationship between cumulative ecological risk and PSU. This result supports the “individual-process-environment” psychological resilience theoretical model, that is, when individuals encounter ecological risks, they will experience stress. At this time, psychological resilience, as an internal resilience factor, helps individuals successfully cope with stress and reduces the possibility of PSU. The result of the current study is consistent with previous study that psychological resilience can buffer the adverse effects of ecological risks on Internet addiction (Huang, 2020).

Second, psychological resilience moderated the relationship between learning goal orientation and PSU. The result also supports the “individual-process-environment” model of psychological resilience. Psychological resilience is a buffer against the stress of achievement goals that can lead to maladjustment (e.g., mobile phone addiction). The finding supports an existing study that individuals can cope with the pressure created by achievement goals and buffer this pressure through psychological resilience (Fergus and Zimmerman, 2005). In addition, learning objectives focus on the ability of individuals to learn new knowledge and improve themselves through assignments, which is manifested by seeking challenging tasks, learning hard even under difficult situations, and treating failures as useful feedback to continue to improve performance (Elliott and Dweck, 1988). Therefore, the level of psychological resilience of individuals with learning goal orientation may be relatively high, and the moderating effect is obvious.

Third, we did not find that psychological resilience moderated the path of the effect of performance goal orientation on PSU. The reason may be that performance goals focus on individuals’ ability to obtain good evaluation or avoid bad evaluation of ability through performance. Individuals with performance goal orientation avoid challenges, decline in performance in the face of obstacles, attribute failure to low ability, and generate bad emotions (Elliott and Dweck, 1988). In addition, studies have shown that individuals with performance goal orientation have a low level of psychological resilience, and when they face failure situations, they are prone to negative emotions, which leads to problematic use behaviors (Fu and Wang, 2019). Therefore, the level of psychological resilience of individuals with performance goal orientation may be relatively low, and the moderating effect is not obvious.

### 4.4 Limitations, future directions, and implications

There are various limitations to the current study that offer promising directions for future research endeavors. Firstly, regarding the mediating mechanisms, our study identified performance goal orientation and learning goal orientation as partial mediators. This suggests that there may be other mediating variables, such as self-regulation or social anxiety, that have not yet been discovered, providing an opportunity for further exploration in follow-up studies. Second, at different stages of college, ecological risk presents developmental characteristics and changes with the continuous interaction between teachers and students (Sang, 2021). The relationship between cumulative ecological risk and PSU may have a developmental trend. Future studies can explore the development and change of cumulative ecological risk and its influence on PSU through longitudinal studies, so as to provide a basis for targeted intervention research. Third, the universities sampled are primarily located in the southern and western regions of China. To enhance the generalizability of the findings, future research should endeavor to expand the selection of universities to encompass a wider geographical range across China.

Despite these limitations, the current study holds significant practical implications. The findings contribute to unraveling the underlying mechanisms of PSU among college students, thereby offering valuable insights for PSU prevention strategies. Key takeaways include the following three points. First, it underscores the crucial role of cumulative ecological risks among college students, emphasizing that these cumulative factors exert a more profound influence on PSU compared to isolated ecological risk factors, so more attention should be paid to various ecological risks. College counselors are needed to actively organize and carry out various activities so that college students can have a strong sense of school belonging, enhance friendship, improve friendship quality and reduce ecological risks. Teachers are also needed to care about students frequently, so that they can feel certain social support. Second, career planning training is needed to let students set learning goals and know what their future goals are at the early stage of college, so as to provide performance goal orientation and learning goal orientation and reduce the possibility of PSU. Third, mental health courses or group counseling activities are needed to improve the psychological resilience of college students, so as to reduce the tendency of PSU.

## 5 Conclusion

In summary, the findings suggest that both performance goal orientation and learning goal orientation serve as intermediaries in the connection between cumulative ecological risk and PSU. Notably, psychological resilience not only moderates the link between cumulative ecological risk and PSU, but also influences the relationship between learning goal orientation and PSU. Consequently, the current study offers practical insights for helping college students enhance their achievement goals and psychological resilience, ultimately leading to a reduction in PSU levels.

## Data Availability

The raw data supporting the conclusions of this article will be made available by the authors, without undue reservation.
